# *piggy*Bac-Based Non-Viral In Vivo Gene Delivery Useful for Production of Genetically Modified Animals and Organs

**DOI:** 10.3390/pharmaceutics12030277

**Published:** 2020-03-19

**Authors:** Masahiro Sato, Emi Inada, Issei Saitoh, Satoshi Watanabe, Shingo Nakamura

**Affiliations:** 1 Section of Gene Expression Regulation, Frontier Science Research Center, Kagoshima University, Kagoshima 890-8544, Japan; 2 Department of Pediatric Dentistry, Graduate School of Medical and Dental Sciences, Kagoshima University, Kagoshima 890-8544, Japan; inada@dent.kagoshima-u.ac.jp; 3 Division of Pediatric Dentistry, Department of Oral Health Sciences, Course for Oral Life Science, Graduate School of Medical and Dental Sciences, Niigata University, Niigata 951-8514, Japan; isaito@dent.niigata-u.ac.jp; 4 Animal Genome Unit, Institute of Livestock and Grassland Science, National Agriculture and Food Research Organization (NARO), 2 Ikenodai, Tsukuba, Ibaraki 305-0901, Japan; kettle@affrc.go.jp; 5 Division of Biomedical Engineering, National Defense Medical College Research Institute, Saitama 359-8513, Japan; snaka@ndmc.ac.jp

**Keywords:** *piggy*Bac, transposon, non-viral gene delivery, electroporation, hydrodynamics, genetically modified animals, gene of interest, long-term gene expression, chromosomal integration

## Abstract

In vivo gene delivery involves direct injection of nucleic acids (NAs) into tissues, organs, or tail-veins. It has been recognized as a useful tool for evaluating the function of a gene of interest (GOI), creating models for human disease and basic research targeting gene therapy. Cargo frequently used for gene delivery are largely divided into viral and non-viral vectors. Viral vectors have strong infectious activity and do not require the use of instruments or reagents helpful for gene delivery but bear immunological and tumorigenic problems. In contrast, non-viral vectors strictly require instruments (i.e., electroporator) or reagents (i.e., liposomes) for enhanced uptake of NAs by cells and are often accompanied by weak transfection activity, with less immunological and tumorigenic problems. Chromosomal integration of GOI-bearing transgenes would be ideal for achieving long-term expression of GOI. *piggy*Bac (PB), one of three transposons (PB, *Sleeping Beauty* (SB), and *Tol2*) found thus far, has been used for efficient transfection of GOI in various mammalian cells in vitro and in vivo. In this review, we outline recent achievements of PB-based production of genetically modified animals and organs and will provide some experimental concepts using this system.

## 1. Introduction

Viral vectors, including adenoviral (AV), adeno-associated viral (AAV), and lentiviral (LV) vectors, are most commonly used for gene delivery experiments in basic research and clinical gene therapy. They can infect a target cell naturally and effectively, with no additional reagent or equipment (for enhancing gene delivery into a cell) required. The main limitation is their immunogenic property (especially for the use of AV vectors) when directly administered in vivo. The maximum cargo size of viral vectors is restricted, hampering gene delivery of larger genes. Furthermore, construction of viral vectors is time-consuming, costly, and requires living cells for their large-scale production, which is also laborious [1]. Non-viral vectors as exemplified by plasmids (carrying a gene expression unit called “transgene”) can overcome some of these limitations [2]. For example, they can carry a large amounts of DNA (up to 100 kb) without sequences that elicit immunogenic reactions when applied in vivo. Plasmid production is simple (only requiring transformation into *E. coli*, growth on antibiotic-containing agar plates or in liquid culture medium, and purification of plasmid DNA) and inexpensive.

Gene delivery using naked plasmid DNA is used for hepatocyte targeted delivery via tail veins using the hydrodynamics gene delivery (HGD) system, which is one of the most common methods for in vivo gene delivery through high-speed injection of large volumes of plasmid DNA-containing solution [3,4]. Unfortunately, the gene delivery rate of this system still appears lower than that of the viral gene delivery system. Notably, in a typical situation where naked plasmid DNA is placed within tissues, delivery inside a cell is rare unless appropriate gene delivery-assisted reagents (i.e., liposomes and nanoparticles) or apparatuses (i.e., electroporator and gene gun) are used [5].

Non-viral vectors carrying transgenes have been applied locally (via direct introduction) or systemically (via tail-vein injection) to modify target cells in situ for evaluating the function of a gene of interest (GOI), creating models for human disease and basic research toward gene therapy [2,6,7]. Despite several attempts to increase transfection efficiency, chromosomal integration of non-viral vectors into the host genome was difficult. Consequently, this may negatively affect the long-term expression of the GOI as a result of the degradation of plasmids present episomally in the cells’ cytoplasm, and/or dilution during cell proliferation. Thus, low transfection and poor integration rates associated with non-viral vectors have been some of the limiting factors for their use in in vivo gene delivery experiments.

Transposons (also called movable genetic elements) are DNA sequences that can move to different locations within a genome and are now recognized as useful tools for non-viral gene delivery into mammalian cells [8]. This gene delivery can be achieved by transporting a transposon carrying a gene of interest (GOI) and a transposase that mediates chromosomal integration of the GOI into cells. Thus, a GOI (i.e., a fluorescent marker expression unit, a small hairpin (sh)RNA expression cassette, or a therapeutic gene construct) cloned between inverted terminal repeat (ITR) sequences of a transposon-based vector can be inserted into host chromosomes in a highly efficient manner. *Sleeping Beauty* (SB) was the first transposon that was proven to be useful for the delivery of GOIs into various types of cells, and recent studies confirm that SB contributes to establish a broad spectrum of genetic engineering purposes, including transgenesis, insertional mutagenesis, and therapeutic somatic gene transfer, both ex vivo and in vivo [9,10]. *piggy*Bac (PB) is one of three transposons (PB, *Tol2*, and SB) found thus far and is widely used in several research fields, including basic biomedical research using experimental animals and human gene therapy [8,11].

**Figure 1:**
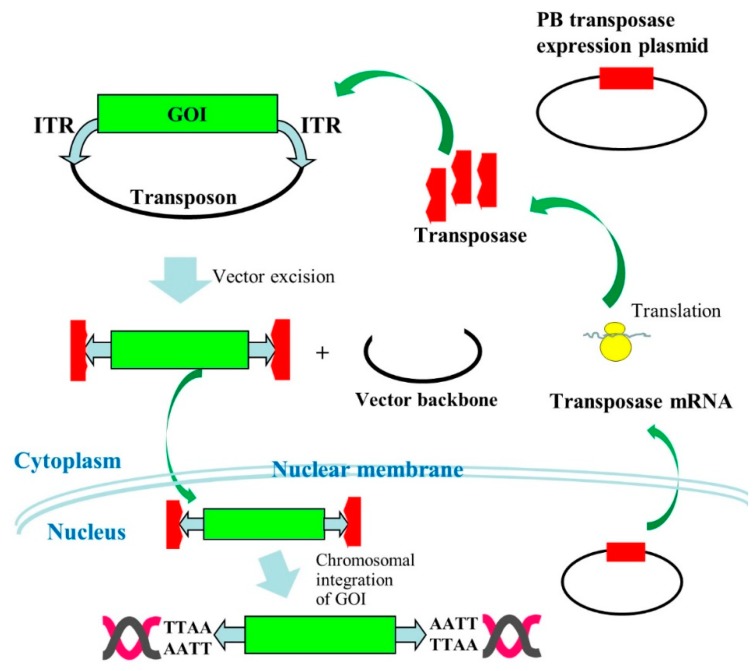
Mechanism of target gene transfer to the host chromosome by the *piggy*Bac (PB) system is described. A gene of interest (GOI) is engineered so as to be surrounded by the inverted terminal repeat (ITR) in a plasmid backbone. When a transposon carrying the GOI is co-transfected with a PB transposase expression vector into a cell, a part of this exogenous DNA is transferred to the nucleus and PB transposase mRNA is synthesized by the PB transposase expression vector. The resulting PB transposase mRNA is next transferred to the cytoplasm where the PB transposase is synthesized using the mRNA as a template. The synthesized PB transposase specifically binds to the ITR in the transposon vector present in cytoplasm and removes the plasmid backbone. The resulting PB transposase/ITR complex is subsequently transferred to the nucleus where chromosomal integration of the GOI via TTAA consensus sequence occurs.

In 1995, Fraser et al. [12] showed for the first time that the PB transposon, which was originally isolated from insect cells, is only active when co-transfected with a PB transposase expression vector. This non-viral, vector-based gene delivery system is extremely simple, as researchers only add a PB transposase expression vector and PB transposon vectors carrying a GOI flanked by two ITR sequences before transfection. When these two components are placed inside a cell, PB transposase generated from the PB transposase expression vector can recognize and bind to transposon-specific ITRs situated at both ends of PB transposon vectors. Subsequently, PB transposase interacts with host chromosomal sites containing the TTAA sequence to enable GOI to be individually integrated via TTAA [13]. This transposon-based chromosomal integration of GOI is called “transposition.” A schematic representation of the PB integration mechanism is shown in Figure 1. Because of chromosomal integration of the GOI after PB-mediated transposition of the transposons, there have been several reports regarding the persistence of long-term gene expression. For instance, Saridey et al. [14] demonstrated that a single injection of plasmid-based PB transposons via the tail vein confers persistent expression of a GOI for ~300 days in liver. Similar results were also provided by other researchers using repeated intravenous injections of PB transposons for ~80 days in liver [15] or intravenous injections of hybrid PB/viral vectors for ~35 weeks in nasal airways [16].

The PB transposon appears to be superior to *Tol2* and SB transposons. This is because PB has a unique property of carrying up to 100-kb transgenes [17,18,19]. Delivery of such large transgenes is impossible with viral vectors. Second, the inserted GOI can be removed by re-introduction of a PB transposase expression vector. This phenomenon is called “seamless” excision, and the resultant cells are thus considered “transgene-free or genetically unmodified cells” [20]. Such chromosomal integration of GOIs and their subsequent removal would be beneficial for researchers to examine the precise role of GOIs in vitro and in vivo. However, in this case, a substantial fraction of the excised transposons often reintegrates into the genome. Li et al. [21] developed a novel PB mutant having an excision activity but not a genome reintegration capability, which is now called “excision-only PB transposase”. A similar observation was also reported by Kesselring et al. [22], who constructed an “excision only” SB transposase through a single amino acid switch in the SB transposase. Thus, DNA transposons, as exemplified by PB, are considered to be promising non-viral alternatives. For example, PB has been employed for a variety of applications, such as in vitro transfection in various mammalian cells, including human [23,24], bovine [25], goat [26], pig [27,28], and mice [14,15,16,29,30]; generation of genome-edited [31] and inducible pluripotent stem (iPS) cells [32,33,34]; generation of transgenic (Tg) animals [35,36,37,38]; and gene discovery via insertional mutagenesis through in vivo gene-wide screening of genes related to neural development and disorders [39]. PB is also known for its importance in inducing immortalization of cultured human deciduous tooth dental pulp cells [40] and hepatocytes in vivo [41].

In the following section, examples of the diverse roles of the PB-based gene delivery system in the production of genetically modified (GM) animals and organs, along with some ideas on possible in vivo PB-based transfection of somatic cells and those useful for increasing PB transposition activity achieved thus far, will be mentioned.

## 2. Diverse Roles of *piggy*Bac (PB)

### 2.1. Systemic Gene Delivery via Tail-Vein Injection of PB

The importance of PB-mediated gene transfer in vivo has frequently been tested using tail-vein injection of a solution containing PB components. Typically, for liver-directed gene delivery, transposon and transposase constructs were administered by HGD, one of the most common methods for in vivo gene delivery through high-speed injection of large volumes of DNA solution [3]. This HGD-based transfection of transposons + PB transposase expression plasmid resulted in sustained GOI expression in the liver and kidney of mice [42]. This HGD-based transfection approach was first developed using mice [3], but has been successfully demonstrated in a broad range of animal models, including rat, rabbit, pig, dog, and monkey [5].

### 2.2. Useful for Regulated Gene Expression In Vivo

The PB system enables inducible gene expression in desired tissue in vivo, soley based on the ability of PB to induce efficient chromosomal integration of GOI in a host cell. For instance, Sariday et al. [14] employed a tetracycline-regulated TetOn system by delivering two genes with one expressing the tetracycline activator and a second element containing the tetracycline response element driving GOI expression (i.e., luciferase cDNA). They performed HGD-mediated tail-vein co-injection of the two PB transposons and PB transposase expression plasmid. In the absence of induction, mice exhibited long-term GOI expression without a detectable leak of expression beyond 120 days, but a several fold increase in expression was achieved when mice were injected intraperitoneally with doxycycline for inducible expression.

**Figure 2:**
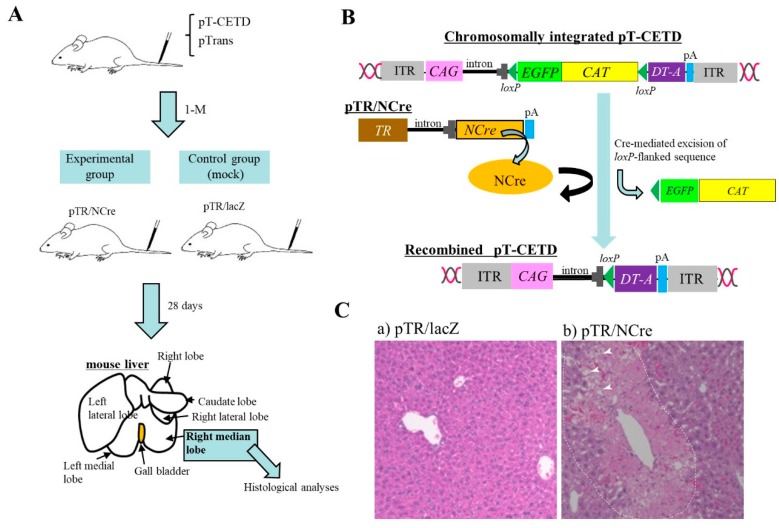
Hydrodynamics-based gene delivery (HGD) and *piggy*Bac (PB) transposon system enable long-term gene expression in murine liver, according to the article by Nakamura et al. [43]. (**A**) Schematic representation of experimental outline. At first, PB transposon (pT-CETD) and PB transposase expression plasmid (pTrans) are co-injected into adult ICR male mice via the tail vein by HGD to perform chromosomal integration of CETD component in hepatocytes. One month later, these mice are intravenously injected with pTR/NCre (shown in **B**) or pTR/lacZ (mock). At 28 days after gene delivery, the right median lobe of liver is dissected for histological analysis. (**B**) Schematic representation of conditional ablation of murine hepatocytes by Cre/*loxP* system. When mice are subjected to HGD with a solution containing pT-CETD and pTrans, chromosomal integration of pT-CETD is thought to occur in some hepatocytes (upper panel). Addition of pTR/NCre to these mice via HGD will elicit the generation of recombined pT-CETD, which in turn generates a toxic protein diphtheria toxin-A chain (DT-A) (lower panel). (**C**) Pathological analysis of pT-CETD-incorporated males assayed 28 days after the second HGD with pTR/lacZ (a) or pTR/NCre (b). Note the presence of focal necrosis (arrowheads in (b); probably caused by conditional expression of DT-A) in an area enclosed by dotted lines. Abbreviations are ITR, inverted terminal repeat; CAG, chicken β-actin-based promoter; EGFP, enhanced green fluorescent protein cDNA; CAT, chloramphenicol acetyltransferase gene; lacZ, gene coding for β-galactosidase; pA, poly(A) sites; TR, transthyretin promoter; NCre, gene coding for Cre with nuclear localization signal.

Nakamura et al. [43] generated a novel mouse model for a hepatic disorder. To induce chromosomal integration of the PB transposon, pT-CETD (carrying a CETD unit (*loxP*-flanked stop cassette, diphtheria toxin-A chain (DT-A) gene, and poly(A) sites) (Figure 2B) in murine hepatocytes, HGD-based tail-vein injection of a TransIT-EE Hydrodynamic Delivery Solution (Takara Bio Inc., Shiga, Japan; hereafter referred to as TransIT-EE) containing pT-CETD + pTrans (PB transposase expresion plasmid) was performed using ICR mice (Figure 2A). Expression of a fluorescent reporter gene was discernible in liver approximately one month after gene delivery, suggesting chromosomal integration of pT-CETD. Thereafter, to induce Cre-mediated excision of floxed sequence in chromosomally integrated pT-CETD, mice, one month after gene delivery of pT-CETD + pTrans, were subjected to tail-vein administration of a plasmid, called pTR/NCre (in which expression of Cre recombinase gene is under the control of a liver-specific promoter; Figure 2B), using TransIT-EE. As a result, these treated mice suffered from liver injury (Figure 2C(b)), probably due to liver-specific expression of a toxic protein DT-A generated from recombined pT-CETD. This experiment suggests that the PB transposon combined with a Cre*/loxP* system is a useful regulatable tool for manipulating hepatocyte function in vivo in non-Tg animals.

### 2.3. Useful for Transgenic (Tg) Animal Production

Pronuclear microinjection (PI) of nucleic acids (NAs) at the zygote stage has been one of the major ways to produce Tg animals since Gordon et al. [44] first developed it. However, it has always been associated with relatively low Tg efficiency (10–40%) [45,46]. Ding et al. [35] first demonstrated that the PB system is useful for increasing the efficiency of murine transgenesis. They performed PI with a solution containing a transposon carrying a visible marker gene coding for red fluorescent protein (RFP) and a PB transposase expression plasmid. Thus, 35% of pups born (62/184) were transgenic. In comparison, only 10% (10/96) of pups were positive when PI was carried out with the transposon donor alone. Li et al. [36] generated Tg rats after PI with a solution containing 30 ng/μL PB plasmid (carrying an RFP gene) and 10 ng/μL transposase expression plasmid. Consequently, 45% of pups born (44/98) were found to be transgenic.

**Figure 3:**
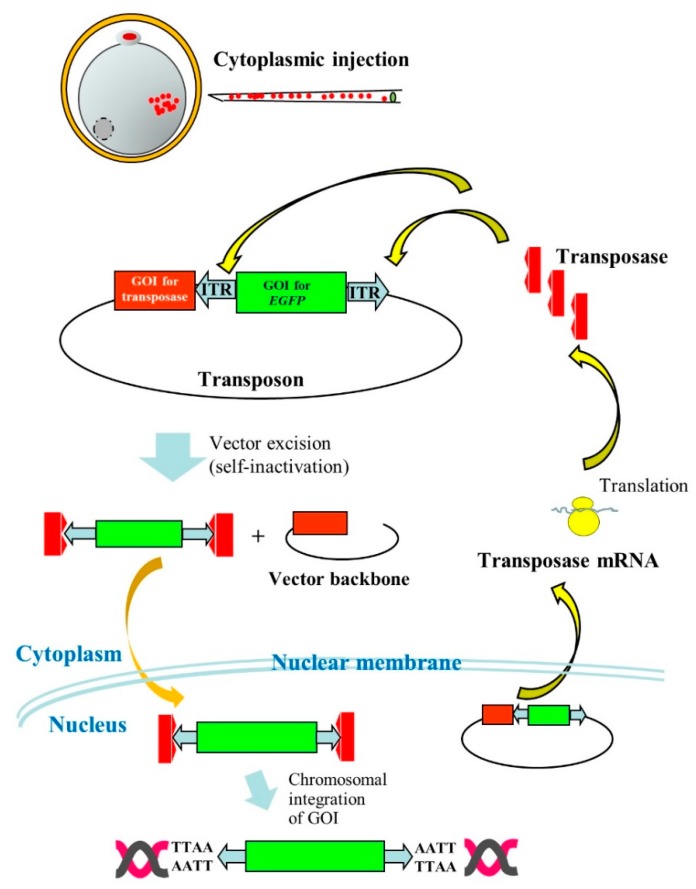
Cytoplasmic injection (CI) of an all-in-one-type *piggy*Bac (PB) vector pmGENIE-3 into porcine parthenogenetically activated oocytes (parthenotes). Following CI of pmGENIE-3, PB transposase mRNA is expressed from the vector and immediately translated into a protein in the cytoplasm. This protein specifically binds to inverted terminal repeat (ITR) present on pmGENIE-3, leading to vector excision. The resulting complex composed of a gene of interest (GOI) for enhanced green fluorescent protein (*EGFP*) cDNA and PB transposase will be subsequently transferred to the nucleus where chromosomal integration of the GOI will occur.

The PB system has also been reported useful for production of Tg domestic animals, such as pigs. In the case of pig zygotes, it is generally hard to visualize pronuclei due to the presence of the lipid droplet layer. Thus, researchers must briefly centrifuge zygotes before microinjection into pronuclei to produce GM pigs [47]. However, this is laborious, as pronuclei are rapidly hindered by the lipid layer within several minutes after centrifugation. Cytoplasmic injection (CI) of DNA appears to be an alternative for the production of Tg animals. Some researchers [48,49] succeeded in creating Tg animals by this procedure, but its success is unstable. This may be due to the ease of delivery of plasmid DNA introduced in cytoplasm of zygotes into nuclei, but rarely integrated into host chromosomes. Li et al. [50] overcame this issue using PB vectors. They performed PI using an all-in-one-type self-inactivating transposon plasmid called pmGENIE-3 (carrying two expression units for transposons and PB transposase) targeting porcine zygotes, and eventually succeeded in producing Tg embryos and piglets. This means that pmGENIE-3 introduced into the cytoplasm of porcine eggs is transferred to nuclei, from which PB transferase mRNA is produced in situ. The resulting mRNA is then transferred to the cytoplasm where the transposase protein is produced. The resultant transposase protein will then bind to ITRs in pmGENIE-3 to generate the transposon/PB transposase complex and cleaved vector backbone. Thereafter, a portion of the former component will be transposed via TTAA present on host chromosomes, as shown in Figure 3.

Tg mice are also produced via the formation of chimeric embryos between normal early mouse embryos (morula or blastocyst) and ES cells that are genetically modified by the PB system [51,52,53]. Rat iPS cells have also been modified by PB to generate Tg rats [54].

Successful production of GM pigs was also demonstrated by somatic cell nuclear transfer (SCNT) of porcine fibroblast cells gene-engineered with the PB system [55].

### 2.4. Focal In Vivo PB Gene Delivery

There are several routes for in vivo gene delivery: one is tail-vein injection of DNA using a needle and the other is local administration of DNA into organs/tissue exposed after surgery using a glass micropipette or needle. In some cases, in vivo electroporation (EP) is applied to the injected site to enhance DNA uptake by cells. Local administration of DNA via intramuscular or intradermal injection is also possible without surgery. In these cases, chromosomal insertion of a transgene via a PB-based gene delivery system may be necessary for persistent expression of the transgene. Furthermore, there is an approach for transplanting gene-engineered cells (which have been stably transfected in vitro) to the target organ or tissue for gene therapeutic purposes; this type of experiment is called an “ex vivo experiment.”

In the following section, several studies concerning in vivo gene delivery are presented.

#### 2.4.1. Gene Delivery to Pancreas

The pancreas is an internal organ having important exocrine and endocrine functions in mammals. Diabetes, pancreatitis, and pancreatic cancer are the most common disorders associated with the pancreas. In vivo gene delivery that targets the pancreas is considered as one of the promising approaches for preventing or curing such diseases as well as exploring the biological functions of genes involved in their pathogenesis.

**Figure 4:**
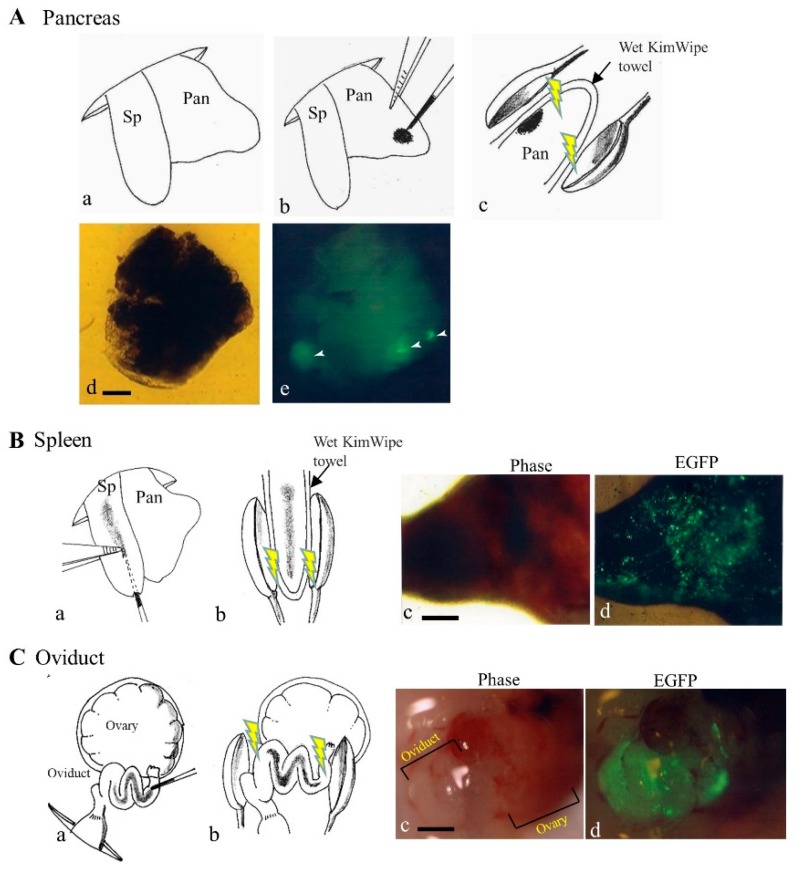
In vivo gene delivery to pancreas (**A**), spleen (**B**), and oviduct (**C**). (**A**) The gene delivery method to pancreatic parenchyma is illustrated by schematic representation (a–c) and photographs (d,e), all of which are derived from our paper [56] under permission of MDPI. Spleen (Sp) and pancreas (Pan) were exposed after dorsal incision of skin and muscle wall under anesthesia (a). Pancreatic parenchyma was injected with a small volume of solution (1−2 μL) containing enhanced green fluorescent protein (EGFP) expression plasmid DNA and Trypan Blue (b). Thereafter, the injected site of the pancreatic parenchyma was subjected to in vivo electroporation (EP) using two tweezer-type electrodes (c). At ~20 days after gene delivery, dissected pancreas still shows EGFP (arrowheads in (e)). Bar: 1 mm. (**B**) The gene delivery method to the spleen is illustrated by schematic representation (a,b) and photographs (c,d). Sp was injected with a solution (~20 μL) containing an EGFP expression plasmid DNA and Trypan Blue (a). Thereafter, the injected site was subjected to in vivo EP using two tweezer-type electrodes (b). At 1 day after gene delivery, the dissected spleen showed EGFP-derived fluorescence (c,d). Bar: 1 mm. (**C**) The gene delivery method to the oviduct is illustrated by schematic representation (a,b) and photographs (c,d). The oviduct was injected with a solution (1−1.5 μL) containing an EGFP expression plasmid DNA and Trypan Blue (a). Thereafter, the entire oviduct was subjected to in vivo EP using two tweezer-type electrodes (b). At 1 day after gene delivery, oviductal epithelium showed EGFP-derived fluorescence (d), but that of untreated intact oviduct did not (c). Bar: 1 mm.

To achieve an efficient gene delivery system targeting pancreatic cells, Sato et al. [56] first employed a non-viral PB-based gene delivery approach coupled with in vivo EP. They injected a solution containing a PB transposon, pT-EGFP (carrying an enhanced green fluorescent protein (EGFP) expression unit), and PB transposase expression plasmid (pTrans) into pancreatic parenchyma of anesthetized adult B6C3F1 female mice under a dissecting microscope with subsequent in vivo EP of the DNA-injected site using tweezer-type electrodes (Figure 4A). Expression of the GOI continued for a minimum of 1.5 months post-gene delivery. The presence of a consensus sequence, TTAA, at the junction between the host chromosomes and transgenes was observed in some samples examined. Sato et al. [56] concluded that such a PB-based gene delivery system could be a useful tool for developing a method to cure diabetes and for exploring other biological applications to assess the function of the pancreas.

#### 2.4.2. Gene Delivery to Spleen

The spleen is an organ found in almost all vertebrates and acts primarily as a blood filter. It plays an important role in the immune system, removing old red blood cells while storing white blood cells. Similar to the gene delivery system that targets pancreatic parenchymal cells, the spleen is accessible for gene delivery on the left flank of an anesthetized mouse after laparotomy, because exogenous plasmid DNA can be easily introduced into the spleen under a dissecting microscope.

Tupin et al. [57] first demonstrated that intrasplenic injection of a solution containing 15 μg of plasmid DNA and subsequent in vivo EP using a square pulse generator resulted in successful transfection of murine splenic cells. We attempted to test whether this in vivo approach of Tupin et al. [57] was useful for efficient acquisition of transfected splenic cells. We directly injected approximately 20 μL of a solution containing a plasmid DNA (i.e., *EGFP*-expressing vector) into an internal portion of mouse spleen (Figure 4B(a)). Immediately after injection, the injected site was subjected to in vivo EP using tweezer-type electrodes (Figure 4B(b)). Consequently, several splenic cells were found be fluorescent (Figure 4B(c,d); unpublished results). Therefore, it is highly anticipated that the gene delivery system of the PB transposon using the current method will be useful for acquisition of stably transfected splenic cells and their persistent expression of the GOIs.

#### 2.4.3. Gene Delivery to Oviducts

The oviduct is a part of the mammalian reproductive system, which is required for the fertilization of ovulated oocytes by sperm and their subsequent transport to the uterus, where embryo implantation occurs. It is known that several biologically active factors secreted from oviductal epithelium play an important role in preimplantation embryo development [58].

A direct gene delivery system that targets oviductal epithelial cells appears to be one of the useful approaches to assess detailed biological roles of these cells. An attempt to transfect oviductal epithelium was first made by Relloso and Esponda [59], who injected a solution containing liposomal-encapsulated DNA into the lumen of an oviduct of adult mice and demonstrated that almost all the mice exhibited gene expression in the oviductal mucosa, although only a few number of cells appears to be transfected. Sato [60] used in vivo EP after intra-oviductal instillation of naked plasmid DNA (i.e., an *EGFP*-expressing vector) to transfect larger numbers of murine oviductal epithelial cells. When we examined this possibility using the same method as that of Sato [60], a large part of the oviductal epithelial cells were found to be successfully (but transiently) transfected, as evidenced by the expression of bright EGFP-derived fluorescence (Figure 4C; unpublished results). It is thus highly expected that gene delivery of the PB transposon system coupled with in vivo EP will enable acquisition of stably transfected oviductal epithelial cells and their long-term expression of the GOIs.

#### 2.4.4. Gene Delivery to Muscle

PB can be applied to basic research towards the establishment of a therapy model targeting Duchenne muscular dystrophy (DMD), which is a lethal muscle-wasting disease that currently does not have a cure and is caused by a mutated dystrophin gene. In this research area, two approaches, namely cell transplantation- and gene-transfer-based approaches, have been employed. The former case involves ex vivo experiments, whereby isolated muscle progenitor cells are gene-engineered to express the full-length dystrophin gene in vitro and these recombinant cells are then transplanted into the muscle tissue of DMD model animals. The latter case involves direct gene delivery to the injured muscle tissue.

For the cell transplantation-based approach, Loperfido et al. [61] suggested the utility of PB for enabling stable expression of the full-length human dystrophin gene in dystrophic mesoangioblasts (MABs), which are precursors to muscle cells. Iyer et al. [62] employed the PB system for long-term expression of full-length dystrophin expression in murine MABs isolated from DMD model mice *mdx/*SCID. When these dystrophin-expressing MABs were transplanted intramuscularly into *mdx/*SCID mice, dystrophin expression occurred in 11%–44% of myofibers in murine muscles and remained stable for the assessed period of 5 months. Furthermore, 80% of fibers showed elasticity properties that were restored to those of wild-type muscles, and transplanted muscles became more resistant to fatigue. This study suggests the possibility that DMD could be cured through autologous cell-based therapeutic approaches using muscle progenitor cells genetically engineered by the PB system.

For the gene-transfer-based approach, Ley et al. [63] performed intramuscular electrotransfer of PB transposons in mice. The tibialis anterior muscles of C57BL/6 female mice were pre-treated with bovine hyaluronidase 2–4 h before plasmid injection using a Hamilton syringe. Direct injection of a solution (30 μL) containing a transposon plasmid and PB transposase expression plasmid was injected intramuscularly under anesthesia. Then, in vivo EP was applied to the DNA-injected muscle site. Unfortunately, Ley et al. [63] failed to achieve sustained transgene expression despite molecular evidence of PB transposition in vivo.

#### 2.4.5. Gene Delivery to Tail

An all-in-one-type plasmid, called mPB-GLuc-mCherry, which confers simultaneous expression of both mCherry (red fluorescent protein) and GLuc (secretory Gaussia luciferase) together with PB transposase, was injected subcutaneously into the tails of mice followed by in vivo EP locally across the injection site [64]. They observed a GLuc signal six months after gene delivery.

#### 2.4.6. Gene Delivery to Bladder

The bladder is an organ that cannot be accessed easily by viral vectors due to the presence of a glycosaminoglycan layer covering the urothelium.

Yu et al. [65] demonstrated that mouse bladder urothelial cells are efficiently, but transiently, transfected with a green fluorescent protein (GFP) expression plasmid DNA when intravesical instillation of the plasmid and subsequent EP targeting the surgically exposed bladder was performed. Bladder-targeted delivery of non-viral DNA combined with EP use would facilitate long-term expression of GOIs in those cells.

#### 2.4.7. Gene Delivery to Brain

Successful delivery of trophic factors to the brain using stem cell-derived neural progenitors is a promising approach to bypass the blood–brain barrier. Akhtar et al. [66] engineered a PB-based doxycycline-regulated vector, which allows inducible and reversible expression of glial cell line-derived neurotrophic factor (GDNF), a protein known to be effective for protection against neurodegenerative diseases, such as Parkinson’s disease. Nucleofection-based gene delivery of this vector enabled the generation of stably transfected human iPS cell-derived neural progenitors. Transplantation of these stably transfected neural progenitors into an adult non-obese diabetic-severe combined immunodeficiency (NOD-SCID) mouse brain and subsequent addition of doxycycline were found to be effective to induce GDNF expression in vivo. These findings support the usefulness of cell-based therapy using gene-engineered stem cells for possible protection against neurodegenerative diseases.

#### 2.4.8. Gene Delivery to Kidney

Insulin-like growth factor-1 receptor (IGF-1R) is known to regulate vascular homeostasis and endothelial function. To examine the role of IGF-1R in oxidative stress-induced endothelial dysfunction, Liang et al. [67] constructed a PB-based vector carrying the IGF-1R gene linked to the vascular endothelial (VE)-cadherin promoter. A solution containing the PB transposon and a PB transposase expression plasmid was injected into the renal vein of a mouse kidney, and the mouse was later subjected to unilateral ureteral obstruction (UUO) to induce interstitial fibrosis and inflammatory cell infiltration. Remarkably, a significant reduction in fibrosis (probably due to IGF-1R overexpression in the kidneys) was observed at Day 7 of UUO. The authors [67] concluded that IGF-1R in the endothelium plays a role in maintaining the endothelial barrier function.

#### 2.4.9. Gene Delivery to Mammary Gland

Mouse mammary glands can regenerate completely by the use of mammary stem cells (MaSCs). To examine the mechanism by which breast cancer develops, Tagaya et al. [68] constructed bacterial artificial chromosome (BAC)-based PB transposons with a vector size of >200 kb to transfect MaSCs via in vitro EP. They transplanted the transfected MaSCs into cleared fat pads of the inguinal mammary glands (from which the endogenous epithelium had been removed) of immune-deficient mice. They observed correct differentiation of the transplanted cells into both basal and luminal cells, as well as milk production after pregnancy. Tagaya et al. [68] also demonstrated that oncogene-induced tumorigenesis is possible when MaSCs transfected with PB transposons, which carry the polyoma-virus middle T antigen gene, are transplanted into mammary glands.

#### 2.4.10. Gene Delivery to Immune Cells

An emerging approach for treating cancer is the programming of circulating T cells with tumor-recognizing capabilities. In other words, circulating T cells could be gene-engineered through in vitro transfection of genes encoding disease-specific chimeric antigen receptors (CARs), so that the resulting recombinant T cells can combat tumor cells once they are reinfused. For practical use, in vitro production of a large number of tumor-specific T cells appears to be difficult. Recently, an efficient method to quickly program circulating T cells with tumor-recognizing capabilities in mice was reported by Smith et al. [69]. Nanoparticles, co-encapsulated with a transposon plasmid (containing leukemia-targeting CAR genes), and a PB transposase expression plasmid were injected intravenously into mice for allowing preferential uptake by circulating T cells. The nanoparticles used were engineered for selective uptake by lymphocytes through receptor-mediated endocytosis, thereby bringing about long-term disease remission. Consequently, the in situ-engineered T cells exhibited similar activity as that of conventionally engineered T cells generated by ex vivo gene transfer. This approach will be useful as a practical and broadly applicable treatment that can generate anti-tumor immunity “on demand” for oncologists in a variety of settings.

It is conceivable that antigen-specific T lymphocytes may be used for long-term expression of therapeutic proteins in vivo. O’Neil et al. [70] engineered CD8+ cells with a murine erythropoietin-expressing transposon, and then these cells were subjected to tail-vein injection in conjunction with a T cell vaccine. Vaccination stimulated long-term T cell engraftment, persistence, and transgene expression, which enabled detection of modified cells up to 300 days after adoptive transfer. Furthermore, the elevated hematocrit continued for more than 20 weeks in the treated mouse. Based on these findings, antigen-specific T lymphocytes may be utilized as a regulatable peptide delivery platform for in vivo therapy.

### 2.5. In Utero Gene Delivery

In utero EP (IUE) is an effective transfection method for delivering plasmid DNA into neural progenitor cells and neurons of mammalian neocortices of fetal brains [71,72,73,74,75], and fetal tissue including the skin [76], lungs [77], and retinal ganglion cells [78] in vivo. However, introduced plasmid DNA present episomally and is often inactivated or lost after cell division, still remains a problem.

To overcome this, researchers [79,80] demonstrated that IUE, using a PB transposase expression plasmid and transposon plasmids with different promoters that allow for shRNA and bicistronic expression, resulted in stable somatic cellular transgenesis of neurons and glia. These experiments revealed that the PB-based IUE method provides a valuable new tool for tracking and manipulating neural lineages. Recently, Lu et al. [39] applied IUE coupled with PB-mediated somatic mutagenesis to identify potential genes involved in the behavior of cortical neurons in the developing neocortex that often lead to malformations of cortical development (MCDs). IUE was performed at Day 14.5 of the embryonic stage, at the time when cortical neurogenesis is known to be most active. Screening was performed based on the inability of these cells to relocate correctly within the cortex in vivo. In a fetal brain subjected to IUE, extensive random insertional mutations are highly expected. When the insertion sites were assessed using splinkerette-PCR, among the 33 candidate MCD genes identified through this screening, several genes had already been implicated in neural development and disorders. Lu et al. [39] concluded that this approach is able to identify potential mouse genes involved in cortical development and MCD pathogenesis.

### 2.6. Application to Gene Therapy

PB can also be efficiently applied for in vivo gene transfer in mice via HGD to correct phenotypes of inherited disease. Liver sinusoidal endothelial cells are a major endogenous source of Factor VIII (FVIII), and the absence of FVIII is known to cause the human congenital bleeding disorder, “hemophilia A”. By correcting hemophilia A-associated phenotypes, Matsui et al. [81] attempted to cure hemophilia A via HGD-based injections of a PB transposon plasmid carrying full-length FVIII cDNA and PB transposase expression plasmid into a hemophilia A mouse model. They observed stable production of circulating FVIII for over 300 days without the development of anti-FVIII antibodies. A similar phenotypic correction of hemophilia A [82] or B [83] was also reported after PB-mediated gene transfer in mouse liver.

As mentioned previously, PB can be applied for therapeutic approaches towards DMD. Cell transplantation-based therapy is always accompanied with heterologous transplantation issues. Recently, the experiments of Iyer et al. [62] suggested the possibility that muscle cell precursors isolated from DMD model mice could be gene-engineered after PB-based transfection with the normal dystrophin gene. If this is realized, autologous transplantation will be possible for curing the dysfunction muscle tissue. In contrast, direct gene-transfer-based therapy has some limitations, as exemplified by transgene silencing issues (mentioned in Section 2.4.4) as well as the difficulty in mediating systemic and sustained dystrophin expression [84].

## 3. Improvement of PB

As mentioned previously, PB has the ability to introduce GOI into host chromosomes via the TTAA sequence. If the PB system is further elaborated, the quality of the PB-mediated transposition process, which is important for persistent expression of GOI, should increase. Several attempts to realize this purpose are shown in the following section.

### 3.1. Super PB Transposase

The activity of PB transposase can be artificially modified to enhance its efficacy in mammalian cells. The first attempt was to engineer the transposase DNA sequence by codon optimization for use in mammalian cells. For instance, mouse and human codon optimization caused increased PB transposition activity several times [85,86]. Later, for further improvement, novel amino acid mutations (generated by using error-prone PCR) were introduced into PB transposase. This improved “hyperactive PB transposase” is named 7pB and carries seven mutations [87]. Doherty et al. [88] tested whether 7pB is effective for increasing the gene delivery rate in vivo using HGD-based tail-vein injection of transposase DNA combined with luciferase reporter transposons. They observed that an approximate 10-fold greater long-term gene expression is achieved in mice.

### 3.2. PB Transposase mRNA

PB-mediated transposition is usually required for co-transfection of a donor plasmid (carrying GOI) and a helper plasmid (carrying the PB transposase gene). The main drawback of this approach may be the persistent presence of transposase at the site co-transfected with those components, as the helper plasmid is often present in an episomal state. This often leads to multiple transposition cycles, which may result in increased potential damage to the chromosome. Thus, employment of a more labile (therefore, showing a short half-life) source of transposase, such as PB transposase mRNA or protein, is desirable. Indeed, efficient transposition using in vitro-transcribed PB transposase mRNAs has been documented in various species including mammals [89,90,91]. Notably, PB transposase mRNA (Improved Super *piggy*Bac transposase mRNA) is now commercially available from Transposagen Biopharmaceuticals, Inc. (#SPB-100; Lexington, KY, USA).

### 3.3. Modification of Inverted Terminal Repeat (ITR)

The ITRs recognized by PB transposase can be engineered to accelerate transposase activity. Zhang et al. [92] trimmed the 5′ and 3′ ITRs without losing their functional region required for PB transposition; thus, leading to the formation of PB vectors with a reduced total size. Alternatively, ITR variants bearing nucleotide substitutions were generated by random PCR. Consequently, the 5′ ITR harboring T53C and C146T mutations demonstrated improved transposition when used in combination with transposase [86].

### 3.4. Use of Insulators

Transposon vectors usually mediate efficient transgene integration, but once integrated into a chromosome, transgenes may undergo epigenetic effects as exemplified by “gene silencing” [93]. One way to avoid gene silencing of a GOI is to include “insulators” into transposon cassettes. Insulators are DNA *cis*-regulatory elements having chromatin boundary and/or enhancer-blocker properties [94]. Expression of a GOI is guaranteed when it is placed between two insulators, which prevents propagation of a silencing chromatin structure over the GOI. Bire et al. [89] demonstrated the effectiveness of insulators for increasing the expression rate of a GOI after PB-based gene delivery.

### 3.5. Use of Epigenetic Regulatory Element

Matrix attachment regions (MARs) are cis-acting DNA elements and known to act as epigenetic regulatory sequences that increase gene expression [95]. Ley et al. [96] demonstrated that incorporation of human MAR 1-68 in a PB transposon cassette was beneficial for reduced silencing effects and caused increased transgene expression in cultured cells. This finding was also confirmed by Zhao et al. (2017) [97]. Transfection of Chinese hamster ovary cells with the constructs carrying GOI flanked by different combinations of human β-interferon and β-globin MAR (iMAR and gMAR, respectively), which was driven by the cytomegalovirus (CMV) or simian virus (SV) 40 promoter, resulted in an increased transfection efficiency and transient expression of GOI expression as well as an increased ratio of stably transfected positive colonies.

### 3.6. Hybrid Non-Viral/Viral Vector System

As mentioned above, the PB-based gene delivery system is used for co-delivery of plasmid-based vectors, namely, PB transposons and PB transposase expression vectors. However, the gene delivery rate is still inefficient in somatic cells in vitro. To improve the low gene delivery rate, Cooney et al. [16] constructed hybrid PB/viral vectors, in which a transposon sequence or expression unit for a gene coding for hyperactive PB transposase (termed iPB7) was inserted into the *E1* region of a first-generation adenoviral vector (Ad5). Nasal delivery of PB/viral vectors (Ad-iPB7 for expression of PB transposase and *piggy*Bac/Ad for expression of GOIs) to mice resulted in persistent expression of GOIs, probably due to the presence of stably transfected cells after overexpression of the hyperactive PB transposase. Consequently, this hybrid vector system could be a promising tool for in vivo gene delivery, by increasing delivery efficiency.

Similarly, Cunningham et al. [98] developed a hybrid recombinant AAV (rAAV)/PB vector system for enabling efficient integration of GOI into the hepatocyte genome. When this vector carrying EGFP expression cassette was delivered into wild-type newborn mice, a 20-fold increase (when compared to the traditional rAAV gene delivery) in the number of stably gene-modified hepatocytes was observed four weeks post-treatment. Furthermore, a single treatment with a hybrid rAAV/PB vector (carrying therapeutic gene expression cassette) to newborn with severe urea cycle defects was sufficient to confer robust and stable phenotype correction.

Progressive familial intrahepatic cholestasis type 3 (PFIC3), an inherited juvenile-onset, rare hereditary cholestatic disorder, is caused by homozygous mutation in the ATP binding cassette subfamily B member 4 (*ABCB4*) gene. Siew et al. [99] constructed a hybrid rAAV/PB vector system for liver-targeted gene therapy and applied this vector through intraperitoneal injection in 20 μL volumes into newborn (>3 days after birth) *ABCB4* KO mice, a murine model of PFIC3. A single dose of the hybrid vector led to life-long restoration of bile composition, prevention of biliary cirrhosis, and a substantial reduction in tumorigenesis. They concluded that this hybrid rAAV/PB transposon vector strategy is powerful for correcting juvenile-onset chronic liver disease and reducing the tumorigenicity of PFIC3.

## 4. Conclusions

Tg animals have been frequently used for overexpression of GOIs in a specific tissue or cell type in vivo, but their production and propagation are time-consuming and costly. PB-based gene delivery system is now recognized as a useful non-viral vector, enabling delivery of larger-sized DNA fragments, long-term expression of the constructed GOI, and its chromosomal integration and subsequent removal. These properties are beneficial for simple and convenient production of non-germline GM model animals (carrying organs or tissue that are stably transfected), which are useful for exploring the mechanisms of human diseases and elucidating the biological functions of newly isolated genes.

The low efficiency of gene delivery is a major issue when non-viral gene delivery is applied in vivo. However, the use of PB in combination with in vivo EP is now being recognized as a promising tool for overcoming this issue, especially in the case of local gene delivery-targeted organs or tissue. Further improvement of the PB system itself is also required for this purpose. The PB/viral hybrid vector system, modification of transposase as exemplified by hyperactive PB transposase and excision-competent, integration-defective transposase, and the use of PB transposase mRNA (and protein if possible) would be some of the candidates to be employed as efficient gene delivery systems.

## Abbreviations

AAV	Adeno-associated viral
ABCB4	ATP binding cassette subfamily B member 4
AV	Adenoviral
BAC	Bacterial artificial chromosome
CAG	Chicken β-actin-based promoter
CARs	Chimeric antigen receptors
CAT	Chloramphenicol acetyltransferase gene
CI	Cytoplasmic injection
CMV	Cytomegalovirus
DMD	Duchenne muscular dystrophy
DT-A	Diphtheria toxin-A chain
EGFP	Enhanced green fluorescent protein
EP	Electroporation
ES	Embryonic stem
FVIII	Factor VIII
GDNF	Glial cell line-derived neurotrophic factor
GFP	Green fluorescent protein
GLuc	Secretory Gaussia luciferase
GM	Genetically modified
gMAR	β-globin MAR
GOI	Gene of interest
HGD	Hydrodynamics gene delivery
IGF-1R	Insulin-like growth factor-1 receptor
iMAR	β-interferon MAR
iPS	Inducible pluripotent stem
ITR	Inverted terminal repeats
IUE	In utero EP
LacZ	Gene coding for β-galactosidase
LV	Lentiviral
MABs	Mesoangioblasts
MAR	Matrix attachment regions
MaSC	Mammary stem cell
MCDs	Malformations of cortical development
mCherry	Red fluorescent protein
NAs	Nucleic acids
NCre	Gene coding for Cre with nuclear localization signal
NOD-SCID	Non-obese diabetic-severe combined immunodeficiency
pA	Poly(A) sites
Pan	Pancreas
PB	*piggy*Bac
PFIC3	Progressive familial intrahepatic cholestasis type 3
PI	Pronuclear microinjection
rAAV	Recombinant AAV
RFP	Red fluorescent protein
SB	*Sleeping Beauty*
SCNT	Somatic cell nuclear transfer
shRNA	Small hairpin RNA
Sp	Spleen
SV	Simian virus
Tg	Transgenic
TR	Transthyretin promoter
UUO	Unilateral ureteral obstruction
VE	Vascular endothelial
